# Role of wild ruminants in the epidemiology of bluetongue virus serotypes 1, 4 and 8 in Spain

**DOI:** 10.1186/1297-9716-42-88

**Published:** 2011-07-23

**Authors:** Ignacio García-Bocanegra, Antonio Arenas-Montes, Cristina Lorca-Oró, Joan Pujols, Miguel Ángel González, Sebastián Napp, Félix Gómez-Guillamón, Irene Zorrilla, Elena San Miguel, Antonio Arenas

**Affiliations:** 1 Departamento de Sanidad Animal. Facultad de Veterinaria, UCO, Campus Universitarios de Rabanales 14071, Córdoba, Spain; 2 Centre de Recerca en Sanitat Animal (CReSA), UAB-IRTA, Campus de la Universitat Autònoma de Barcelona, 08193 Bellaterra, Barcelona, Spain; 3 Laboratorio de Producción Animal. Consejería de Agricultura y Pesca, Sevilla, Spain; 4 Consejería de Medio Ambiente, Junta de Andalucía, 29010, Málaga, Spain; 5 Centro de Análisis y Diagnóstico de la Fauna Silvestre (CAD), Conserjería de Medio Ambiente (EGMASA), Junta de Andalucía, Málaga, Spain; 6 Laboratorio Central de Veterinaria, Algete (Madrid), Spain

## Abstract

Although the importance of wild ruminants as potential reservoirs of bluetongue virus (BTV) has been suggested, the role played by these species in the epidemiology of BT in Europe is still unclear. We carried out a serologic and virologic survey to assess the role of wild ruminants in the transmission and maintenance of BTV in Andalusia (southern Spain) between 2006 and 2010.

A total of 473 out of 1339 (35.3%) wild ruminants analyzed showed antibodies against BTV by both ELISA and serum neutralization test (SNT). The presence of neutralizing antibodies to BTV-1 and BTV-4 were detected in the four species analyzed (red deer, roe deer, fallow deer and mouflon), while seropositivity against BTV-8 was found in red deer, fallow deer and mouflon but not in roe deer. Statistically significant differences were found among species, ages and sampling regions. BTV RNA was detected in twenty-one out of 1013 wild ruminants (2.1%) tested. BTV-1 and BTV-4 RNA were confirmed in red deer and mouflon by specific rRT-PCR.

BTV-1 and BTV-4 seropositive and RNA positive wild ruminants, including juveniles and sub-adults, were detected years after the last outbreak was reported in livestock. In addition, between the 2008/2009 and the 2010/2011 hunting seasons, the seroprevalence against BTV-1, BTV-4 and BTV-8 increased in the majority of provinces, and these serotypes were detected in many areas where BTV outbreaks were not reported in domestic ruminants. The results indicate that wild ruminants seem to be implicated in the dissemination and persistence of BTV in Spain.

## Introduction

Bluetongue (BT) is a vector-borne disease caused by a group of viruses belonging to the genus *Orbivirus*, which is mainly transmitted between vertebrate hosts by biting midges of the genus *Culicoides*. To date, 24 distinct BT virus (BTV) serotypes have been identified; three of which were found in Andalusia (southern Spain) during the last decade. In October 2004, BTV-4 was detected in Cádiz Province (south-west Andalusia), and a total of 316 outbreaks were reported in livestock. The last BTV-4 outbreak was detected in October 2005, and the country was declared free of BTV-4 in March 2009 [1]. However, in October 2010 a new BTV-4 outbreak was detected in Cádiz, and until December 2010 eight further outbreaks were reported.

In July 2007, a new BT epidemic, caused by BTV-1, affected southern Spain. BTV-1 causes clinical disease to both sheep and goats and 4446 outbreaks were detected from July 2007 through December 2008 in Andalusia. While no BTV-1 outbreaks were detected during 2009, two new cases were confirmed at the end of 2010 in the Jaén Province (northern Andalusia) [2]. Furthermore, BTV-8 was detected in northern Spain in January 2008, and arrived in Andalusia in October 2008. Until March 2009, 23 BTV-8 outbreaks were reported, followed by a period without apparent cases. In November 2010, a new BTV-8 outbreak was confirmed.

A mandatory vaccination program in domestic ruminants against serotype 4 started in 2004 and was maintained until 2008, two years after the last outbreak was reported. In 2010 vaccination against BTV-4 was resumed in the southern provinces of Andalusia to prevent introduction from northern Africa [3]. Since November 2006, animals have also been vaccinated against serotype 1, and since the end of 2008 also against BTV-8. Currently, BTV-1, BTV-4 and BTV-8 are endemic in Andalusia, and therefore the southern regions of Spain are considered restriction zones for these serotypes [2]. A live attenuated vaccine against BTV-4 was used between 2004 and 2005. However, since 2006 the commercial vaccines against the three serotypes are inactivated vaccines [3].

A wide range of wild ruminant species in Europe have been affected by BTV [4-6]. In Spain, seropositivity to BTV was found in central and southern regions [7,8]. In addition, clinically affected mouflons (*Ovis aries*) were reported in Andalusia [9,10].

The distribution and density of wild ungulates in Spain have increased in recent decades and their populations usually share pastures with domestic ruminants [11]. Although the role played by wild ruminant species in the epidemiology of BT in Europe is still unclear, the importance of these species as both potential reservoirs and sentinels of BTV has been suggested, especially in regions where livestock and wild ruminants share the same habitat [6,10,12-14]. In order to investigate the possible role of wild ruminants in the epidemiology of the bluetongue virus, a large-scale serologic and virologic survey was carried out in southern Spain from 2006 through 2010.

## Materials and methods

### Sampling

**Figure 1:**
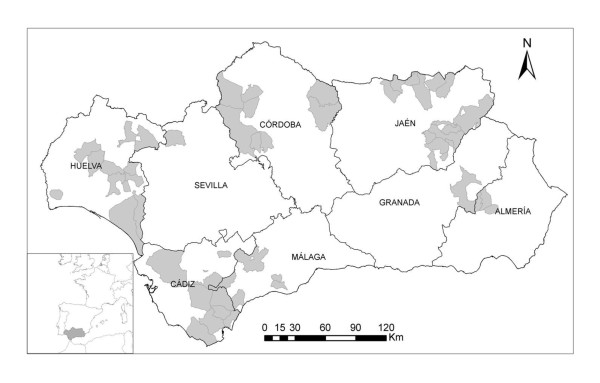
**Map showing the location of Andalusia (southern Spain) and the provinces in which it is divided**. Grey areas indicate the municipalities in which samples from wild ruminants were collected during the study period, 2006-2010. Sampling regions were grouped in western (including the Provinces of Huelva, Cádiz and Seville), central (including the Provinces of Córdoba and Málaga) and eastern regions (including the Provinces of Granada, Jaén and Almería).

**Figure 2:**
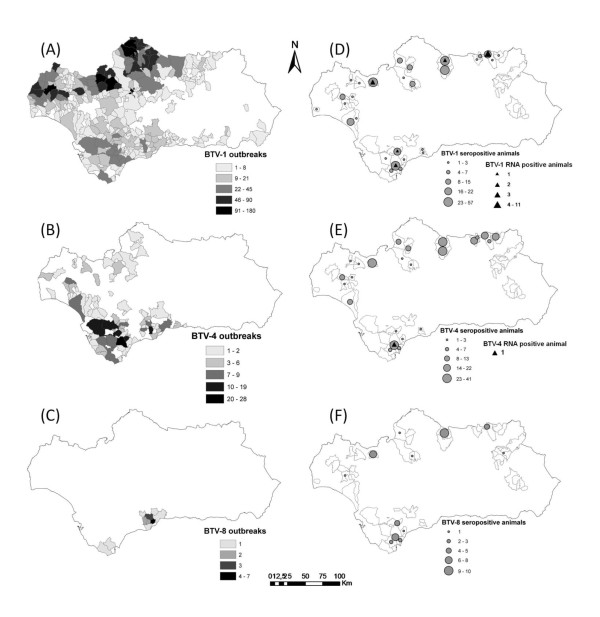
**Map of Andalusia (southern Spain) showing the spatial distribution of BTV serotypes in livestock and wild ruminants, period 2006-2010**. The gradient of grey indicates the number of BTV-1 (A), BTV-4 (B) and BTV-8 (C) infected farms by domestic ruminants. Grey dots size is proportional to the number of BTV-1 (D), BTV-4 (E) and BTV-8 (F) seropositive wild ruminants detected and the black triangles indicate the presence of BTV-1 RNA (D) and BTV-4 RNA (E) positive animals. White areas indicate the regions in which wild ruminants were sampled.

The study was conducted in Andalusia, located in southern Spain (36° N - 38° 60' N, 1° 75' W - 7° 25' W) (Figure 1) from 2006 through 2010, a region and time period with a wide circulation of BTV in livestock (Figure 2).

Post-mortem examinations were carried out on 1446 randomly selected free-ranging wild ruminants shot during the hunting seasons (October-March) of the five study years. A total of 1396 blood samples were collected into sterile tubes without anticoagulant and centrifuged at 400 *g *for 15 min. Blood samples from 336 animals were also placed in sterile tubes containing EDTA for RT-PCR analysis. In addition, 759 spleen samples were collected during the necropsies. All samples were stored at -20°C until analysis. Species, location, age, sex and hunting season of each animal were recorded. The animals were classified into three age groups based on tooth replacement: juveniles (< 1 year old), sub-adults (between 1 and 2 years old) and adults (> 2 years old). Unfortunately, the ages of 327 animals and the sexes of 382 could not be recorded. Data on BTV outbreaks in domestic animals in Andalusia were obtained from the Spanish Ministry of Agriculture, Fisheries and Food [2].

### Serological analyses

The presence of antibodies against the VP7 protein of BTV was determined using a commercial double-antigen enzyme linked assay (ELISA; INGEZIM BTV DR 12.BTV.K0, INGENASA, Madrid, Spain) according to the manufacturer's recommendations. This ELISA has been used in previous surveys in wild ruminants [8,15].

ELISA positive sera were then tested with the serum neutralization test (SNT) for BTV-1, BTV-4 and BTV-8 as previously described [16]. Briefly, serum samples were inactivated at 56°C for 30 min prior to analysis. Sera were diluted (1:2 - 1:256) in MEM (Eagle's minimum essential medium) and mixed with 100 TCID_50 _(50% tissue culture infective doses) of each reference strain, BTV-1, BTV-4 and BTV-8. Plates were incubated for 1 h 30 min at 37°C. Finally, 100 μL of a Vero E6 cell suspension (1.5 × 10^4 ^cells/well) were added in cell growth media (MEM supplemented with 15% fetal calf serum, 300 μg L-glutamine/mL, 300 U penicillin/mL and 300 μg streptomycin/mL). The mixture was further incubated for 6 - 7 days at 37 μC until a cytopathic effect (CPE) was developed in control wells containing 100 TCID_50 _of virus and no serum. Only samples that showed neutralization (absence of CPE) at dilutions ≥1:4 were considered positive [15]. Controls for cytotoxicity in the absence of virus were included for each sample at a dilution of 1:2. Seroprevalence to BTV was determined from samples positive by ELISA and SNT.

### Virological analyses

Blood and spleen samples were analyzed using a semi-quantitative real time reverse transcriptase-PCR (rRT-PCR) detecting a conserved region within segment 5 of the BTV genome [17]. Serotype specific rRT-PCR was performed on BTV positive samples, according to the following methods: for BTV-1 [18]; for BTV-4 [Agüero, not published]; for BTV-8 [19]. Field-isolated strains were used as positive controls for PCR amplification: BTV-1 (BTV-1 SPA/2007/01), BTV-4 (BTV-4 isolated in Cádiz in 2004) and BTV-8 (BTV-8 BEL/2006/01).

### Statistical analyses

Association between explanatory variables (species, age, region, sex and hunting season) and BTV seropositivity was tested in three steps. Firstly, a bivariate chi-square test was performed to obtain an indication of the relevance of the explanatory variables in the risk of an animal being seropositive. Secondly, factors showing a *p*-value < 0.25 were further scrutinized for associations using the Pearson correlation coefficient (*r*) to avoid collinearity problems. If *r *was larger than 0.4, the variable more clearly linked to BTV infection was retained. The third step involved a multiple logistic-regression model as described by Hosmer and Lemeshow [20]. This was performed using a non-automatic backward selection of variables. Biologically plausible confounding factors were tested using Mantel-Haenszel analysis and confounding was considered to be potentially significant if odds ratio (OR) shifted appreciably. Changes in the OR greater than 30% were considered indicative of confounding. Potential two-way interactions between the variables were tested for significance in the model. The model was re-run until all the remaining variables presented statistically significant values (likelihood-ratio Wald test, *P *< 0.05), and a potential causal relationship with the response variable existed. Statistical analyses were performed using SPSS 15.0 (Statistical Package for Social Sciences (SPSS) Inc., Chicago, IL, USA).

## Results

A total of 639 out of 1396 wild ruminants (45.8%) analyzed showed antibodies against BTV by ELISA. Fifty-seven serum samples positive by ELISA could not be analyzed by SNT due to cytotoxicity, and 109 samples were positive by ELISA but negative by SNT. Therefore, a total of 473 out of 1339 wild ruminants (35.3%; CI_95%_: 32.7-37.9) analyzed showed antibodies against BTV by both ELISA and SNT. Seroprevalences to BTV serotypes were 25.6% to BTV-1 (343/1339; CI_95%_: 23.3-27.9), 21.6% to BTV-4 (289/1339; CI_95%_: 19.4-23.8) and 3.4% to BTV-8 (45/1339; CI_95%_: 2.4-4-4).

Seroprevalence among species, age classes, regions, sexes, and hunting seasons are shown in Table 1. Although antibodies to BTV were detected in the different species analyzed, the seroprevalence was significantly higher in red deer, fallow deer and mouflon than in roe deer. Statistically significant differences between fallow deer and mouflon were not observed (Table 2). The presence of neutralizing antibodies against BTV-1 and BTV-4 were detected in the four species analyzed, while seropositivity against BTV-8 was found in red deer, fallow deer and mouflon but not in roe deer. Antibodies against both BTV-1 and BTV-4 were detected in 145 animals of the four species analyzed. Nine animals, including red deer, fallow deer and mouflon showed antibodies against both BTV-1 and BTV-8. Seropositivity to BTV-4 and BTV-8 was detected in four red deer and two fallow deer. Twenty-two individuals, including red deer, fallow deer and mouflon showed antibodies against the three virus serotypes.

**Table 1:** Frequency of antibodies against BTV in wild ruminants.

Category	Value	No. Samples*	No. Positive (%)
**Species**			
	Red deer	900	381 (42.3)
	Fallow deer	188	61 (32.4)
	Mouflon	101	28 (27.7)
	Roe deer	150	3 (2.0)

**Age**			
	Juveniles	98	21 (21.4)
	Sub-adults	261	74 (28.4)
	Adults	653	289 (44.3)

Region			
	Western	553	190 (34.4)
	Central	430	196 (45.6)
	Eastern	356	87 (24.4)

**Sex**			
	Female	419	167 (39.9)
	Male	538	232 (43.1)

**Hunting season**			
	2006/2007	51	24 (47.1)
	2007/2008	54	23 (42.6)
	2008/2009	174	41 (23.6)
	2009/2010	744	255 (34.3)
	2010/2011	316	130 (41.1)

*Samples with no data on age or sex and cytotoxic sera were omitted.

**Table 2:** Logistic regression analysis of potential risk factors associated with BTV seropositivity.

Variable	Category	*β*	*P*-value	OR	95% CI
Species	Red deer	0.78	0.04	2.18	(1.28-3.70)
	Roe deer	-2.80	0.00	0.06	(0.02-0.21)
	Fallow deer	0.13	0.67	1.14	(0.63-2.08)
	Mouflon	*	*	*	*
Age	Adults	0.18	0.00	1.92	(1.53-2.40)
	Sub-adults	*	*	*	*
	Juveniles	-0.49	0.10	0.61	(0.34-1.10)
Region	Western	0.83	0.00	2.30	(1.59-3.34)
	Central	1.35	0.00	3.87	(2.65-5.66)
	Eastern	*	*	*	*

*Reference category.

Adult animals presented significantly higher seroprevalence as compared to subadults and juveniles (Table 2). A significantly higher seropositivity was also found in western and central regions as compared to eastern regions (Figure 2). Distribution of BTV serotypes by hunting seasons and provinces are shown in Table 3. Seropositivity to BTV-4 was detected for all the years of sampling, neutralizing antibodies against BTV-1 were found since the 2006/2007 hunting season, while the presence of BTV-8 seropositive animals was confirmed since the 2008/2009 hunting season. BTV seroprevalence did not differ significantly between hunting season and sex.

**Table 3:** Distribution of BTV serotypes by hunting season and province.

	Hunting season				
	
Province	2006/2007	2007/2008	2008/2009	2009/2010	2010/2011
**Almería**	No samples	No samples	No samples	Negative	Negative
**Cádiz**	No samples	Negative	BTV1	BTV1*/BTV4/BTV8	BTV1/BTV4*/BTV8
**Córdoba**	No samples	No samples	BTV1/BTV4	BTV1/BTV4/BTV8	BTV1*/BTV4/BTV8
**Granada**	No samples	No samples	No samples	Negative	Negative
**Huelva**	No samples	No samples	No samples	BTV1/BTV4/BTV8	BTV1/BTV4
**Jaén**	BTV4	BTV1/BTV4	BTV1/BTV4	BTV1/BTV4/BTV8	BTV1*/BTV4/BTV8
**Málaga**	No samples	BTV1*/BTV4	BTV1	BTV1/BTV4/BTV8	Negative
**Sevilla**	BTV4	BTV1*/BTV4	BTV1/BTV4/BTV8	BTV1/BTV4/BTV8	No samples

Presence of positive animals as determined by BTV serotype-specific rRT-PCR.

BTV RNA was detected in twenty-one out of 1013 wild ruminants analyzed (2.1%; CI_95%_: 1.2-2.6). The threshold cycle (Ct) RT-PCR values ranged between 30.1 and 38.1 (median = 35.5). Nineteen red deer and one mouflon were positive by BTV-1 specific rRT-PCR. BTV-1 RNA-positive results were found in the 2006/2007, the 2009/2010 and the 2010/2011 hunting seasons. One spleen sample from an adult deer sampled in the 2010/2011 hunting season was positive by BTV-4 specific rRT-PCR. No pathological lesions compatible with BT were observed in any of the BTV RNA-positive animals.

## Discussion

Our findings confirm that wild ruminants were actively exposed to BTV in the study area. A large number of the samples tested were positive by ELISA but negative by SNT. The differences between the tests, likely reflect that ELISA and SNT measure distinct antibody populations. The ELISA used in the present study detects both IgM and IgG antibodies to BTV, and it has been proven to be highly sensitive and specific in livestock [21]. Even though the quality of sera may also influence the results of both tests, it is particularly important for SNT. In fact, a high number of samples positive by ELISA could not be analyzed by SNT due to cytotoxicity. BTV seroprevalence was determined only from samples positive by both ELISA and SNT and therefore could have been underestimated.

To our knowledge, this is the first study on BTV serotypes in different wild ruminant species. The seroprevalence levels indicate widespread circulation of BTV in red deer, fallow deer and mouflon, which is in agreement with what was previously reported [6-8]. The results support the idea that sero-surveillance on these species would be useful to detect virus circulation, especially in areas where a vaccination program has been implemented in livestock [9,10]. Furthermore, the low seropositivity detected in roe deer is in agreement with previous studies [7,12,22], suggesting that it is not a relevant species in the dissemination of BTV. Differences among species might be related to the natural resistance of the hosts, population densities, geographical distribution, sampling period or management factors.

The higher seroprevalence in adult animals was probably due to a greater exposure of this age group to the virus over time [8,10,12]. Interestingly, seropositivity was found in young animals (juveniles and sub-adults) sampled long after any previous outbreak in domestic ruminants. This was particularly clear in the case of BTV-4: even though the last outbreak in domestic ruminants was reported in October 2005, juveniles and sub-adults of the different wild ruminant species analyzed presented antibodies against BTV-4 between the 2008/2009 and the 2010/2011 hunting seasons in all the provinces where outbreaks had been reported in livestock. Similar findings were observed for BTV-1 and BTV-8, and evidence the ability of BTV to circulate despite no cases reported in domestic ruminants. Whether that circulation indicates the maintenance of BTV within the wild ruminant population despite vaccination of domestic ruminants, or is the consequence of repeated introductions of the virus, merits further studies.

No antibodies against BTV-4, BTV-1 or BTV-8 were detected in wild ruminants sampled prior to the detection of the first outbreak of each serotype in livestock. Between the 2008/2009 and the 2010/2011 hunting seasons, seroprevalence against the different serotypes increased in the majority of areas, even when the same species and ages were compared. These findings could indicate the persistence of antibodies against BTV for very long periods or, more likely, a longer BTV maintenance and circulation in wild ruminants compared to livestock. The lower seropositivity against BTV-8 (3.4%) was not unexpected taking into account that only 24 outbreaks had been reported in livestock in the study area [2].

The risk of being a seropositive animal was 2.3 and 3.9 times higher in the western and central regions, respectively, as compared to the eastern, which is in accordance with the geographical distribution of BTV observed in domestic ruminants (Figure 2). Vector density, host density and environmental factors are possibly implicated in the spatial distribution of BTV [23,24]. However, BTV-1, BTV-4 and BTV-8 seropositive animals were detected in regions where these serotypes were not reported in livestock. This was particularly evident for BTV-8 (Figure 2) and confirms a different spatial distribution of BTV in wild ruminants as compared to livestock [8,15].

The presence of BTV-1 and BTV-4 RNA in red deer and mouflon confirms the susceptibility of these species to BTV infection [9,10]. In addition, the low cycles (Ct) RT-PCR values obtained in 12 animals (Ct lower than 35) support the potential reservoir role of these species. Three of BTV-1 RNA positive wild ruminants were found in November 2009, even though the last BTV-1 outbreak in livestock was reported in December 2008. One of these BTV-1 RNA positive animals showed no antibodies by ELISA or SNT, which could suggest a fresh infection in this animal [12,13]. Furthermore, BTV-1 RNA positive wild ruminants sampled in the 2010/2011 hunting season were detected in locations different from those where the last BTV-1 outbreak was reported in livestock [2]. The BTV-4 RNA positive animal was detected on November 2010, one month after this serotype was first detected on a sentinel farm located at 60 km distance.

Vaccination has been suggested as one of the most important control measures for BT [25]. In this sense, the limited number of cases reported in livestock despite the circulation observed in wild ruminants suggests that vaccination seems to be effective to prevent the clinical disease. However, to prevent outbreaks in livestock, the vaccination of the domestic population would have to be maintained until virus circulation within the wild ruminant population has ceased.

Our results confirm that wild ruminant populations from southern Spain were exposed to BTV-1, BTV4 and BTV-8. The high seroprevalence to BTV-1 and BTV-4 found in the present study, the detection of both BTV seropositive and RNA positive animals, including juvenile animals, years after the last outbreak was reported in livestock, and the presence of the different BTV serotypes in areas where BTV outbreaks had never been reported in domestic ruminants, indicate that wild ruminants seem to be implicated in the dissemination and persistence of BTV, and probably play a significant role as reservoirs for BTV.

## Competing interests

The authors declare that they have no competing interests.

## Authors' contributions

IG, AAM, CL, JP, AA conceived and designed the study and participated in its coordination. IG, AAM, FG, IZ participated in sampling and field work. IG, AAM, CL, JP, MG, IZ, ES carried out the laboratory work. IG, JP, SN, AA analyzed the data. IG, CL, JP, MG, SN, AA drafted the manuscript. All authors read and approved the final manuscript.
